# Extracellular vesicles as minimally invasive biomarkers and therapeutic platforms in rare neurological diseases

**DOI:** 10.3389/fragi.2026.1813553

**Published:** 2026-06-24

**Authors:** Nathan D. Phan, Raghu Rai Sharma, Subbaya Subramanian, Reena V. Kartha

**Affiliations:** 1 Center for Orphan Drug Research, Department of Experimental and Clinical Pharmacology, College of Pharmacy, University of Minnesota, Minneapolis, MN, United States; 2 Department of Surgery, Medical School, University of Minnesota, Minneapolis, MN, United States

**Keywords:** aging, extracellular vesicles, minimally invasive biomarkers, rare neurological diseases, therapeutic applications

## Abstract

Rare neurological diseases (RND) represent a growing but underrecognized global health burden, particularly in aging populations in whom clinical manifestations appear later in life, resulting in substantial morbidity, reduced quality of life, and increased mortality. Advances in understanding and treating these diseases have been hindered by low prevalence, phenotypic heterogeneity, and complex molecular mechanisms. Importantly, shared genetic and mechanistic links between rare and common neurodegenerative disorders, such as *GBA1 (Glucosylceramidase Beta 1)* gene variant-associated Gaucher disease (GD) and Parkinson’s disease (PD), highlight convergent biological pathways that may be leveraged for biomarker discovery and therapeutic innovation. Notably, individuals heterozygous for pathogenic *GBA1* variants, historically considered asymptomatic carriers, are now recognized to have an increased lifetime risk of developing PD and related synucleinopathies. This emerging evidence indicates that even single-allele variants can confer long-term neurological risk, reinforcing the continuum between rare monogenic disorders and more common neurodegenerative diseases. Extracellular Vesicles (EVs) have emerged as a promising, minimally invasive platform for advancing RND research, although translation remains limited by source specificity, vesicle heterogeneity, and incomplete clinical validation. By encapsulating proteins, lipids, and nucleic acids that reflect their cellular origin and disease state, EVs offer unique opportunities for early diagnosis, disease stratification, longitudinal monitoring, and assessment of treatment responses. In this review, we summarize fundamental aspects of EV biology and critically evaluate recent advances in EV-based biomarker discovery for RND, informed by translational insights from more prevalent neurodegenerative conditions. We further discuss the therapeutic potential of EVs, emphasizing their intrinsic biocompatibility, their reported capacity in selected preclinical settings to traverse biological barriers, including the blood-brain barrier, and their versatility as carriers for neuroprotective and gene-modifying cargo. Finally, we address key technical and translational challenges, including isolation, characterization, scalability, and regulatory considerations that currently limit clinical adoption. Collectively, this review highlights emerging opportunities and outlines an integrative translational perspective for EV-based diagnostic and therapeutic strategies into research and clinical paradigms for aging individuals affected by RND.

## 1 Introduction

Rare neurological diseases (RND), including lysosomal storage disorders, leukodystrophies, and inherited neurogenetic conditions, are individually uncommon but collectively affect millions of individuals worldwide. Advances in diagnosis and disease-modifying therapies have significantly extended patient survival, resulting in a growing population of individuals living with lifelong neurological diseases (Pastores and Maegawa, 2013). This growing population faces a substantial and sustained disease burden, with consequences extending beyond affected individuals to families and healthcare systems responsible for long-term care. Many RND present early in life and are characterized by progressive neurological decline, disability, and, in some cases, premature mortality if not intervened upon early (Toze et al., 2021). As survival improves with therapeutic advances and patients age, there is an increasing need for robust, sensitive biomarkers to monitor disease progression, treatment response, and age-related complications. However, reliable biomarkers and effective long-term therapeutic strategies remain limited, underscoring the urgent need for innovative diagnostic and therapeutic approaches (Delaye et al., 2022; Wang S. et al., 2024).

In this review, RND primarily refers to inherited neurological disorders with low population prevalence. These disorders involve a substantial lifelong neurodegenerative or neurometabolic burden. We focus on lysosomal storage disorders, leukodystrophies, and related inborn errors of metabolism. We prioritize disorders where survival into older age, progressive CNS involvement, or emerging neurodegenerative overlap create a need for longitudinal biomarker strategies. Acquired neurological disorders and rare non-CNS conditions were not a primary focus unless used as translational comparators. The literature synthesis was narrative, but guided by a focused search for studies published primarily between 2010 and 2025. Search terms included combinations such as “extracellular vesicles,” “small EVs,” “rare neurological disease,” “lysosomal storage disorder,” “brain-derived EV,” “aging,” and “biomarker.” Emphasis was placed on peer-reviewed original studies, case reports, and consensus guidance relevant to EV characterization, CNS biomarker development, and applications in RND. Preprints were excluded unless their findings demonstrated clear translational relevance and were consistent with the emerging consensus in the field.

Individuals with inborn errors of metabolism (IEMs) constitute a heterogeneous subgroup of RND that often present with neurological and neurodevelopmental involvement. Historically, many IEMs were associated with markedly reduced life expectancy (Driesen and Witters, 2022). Earlier diagnosis through newborn screening, combined with therapeutic advances, has enabled many affected individuals to survive into adulthood and older age, creating an unprecedented convergence between lifelong neuro-metabolic disorders and aging-related conditions. Consequently, clinicians are increasingly encountering aging individuals with RND who experience both chronic disease–related pathology and late-onset neurological comorbidities, including cognitive impairment, neuropathic pain, dementia, and other age-associated complications (Zhou et al., 2024; Moio et al. 2025). In parallel, accumulating evidence suggests that individuals heterozygous for certain IEM-associated genes may not remain entirely asymptomatic across their lifespan. For example, several studies have demonstrated an increased risk of dementia and other neurodegenerative manifestations in individuals with heterozygous *NPC1* mutations, supporting the concept that even single-allele variants can increase age-dependent neurological vulnerability (Lopergolo et al., 2025). This emerging clinical landscape highlights the need to understand how lifelong genetic and metabolic dysfunction intersects with fundamental aging biology to optimize care across the lifespan.

Extracellular Vesicles (EVs) have emerged as promising candidates to fill this critical knowledge gap. EVs are central mediators of intercellular communication and carry bioactive cargo that reflects the physiological and pathological state of their cells of origin (Raposo and Stoorvogel, 2013; Doyle and Wang, 2019). Growing evidence from studies of common neurodegenerative diseases demonstrates that EVs can provide insights into disease mechanisms, facilitate biomarker discovery, and support therapeutic development (Doyle and Wang, 2019). However, EV research has disproportionately focused on prevalent disorders such as Alzheimer’s disease (AD) and Parkinson’s disease (PD), leaving their roles in RND comparatively underexplored. This gap is particularly striking given the rarity, heterogeneity, and early-onset of neurodegeneration in RND, as EV-based liquid biopsy approaches offer a uniquely adaptable platform for advancing mechanistic insight, developing minimally invasive biomarkers, and enabling targeted therapeutic delivery in settings where access to central nervous system tissue and opportunities for longitudinal sampling are limited (Colombo et al., 2014; Kalluri and LeBleu, 2020). Therefore, in this review, we summarize the current knowledge of EV biology and its role in RND. This critically evaluated their diagnostic and therapeutic potential, discussed technical and translational challenges, and highlighted future directions for integrating EV-based strategies into precision medicine approaches for aging individuals affected by RND.

## 2 Overview of extracellular vesicles

EVs are nanoscale, membrane-bound particles secreted by virtually all cell types, serving as critical mediators of intercellular communication and modulators of physiological and pathological processes (Raposo and Stoorvogel, 2013; Kumar A. et al., 2024). EVs transport diverse molecular cargo, including proteins, lipids, and nucleic acids, that reflect the molecular and functional state of their cells of origin, positioning them as promising candidates for diagnostic biomarkers and therapeutic delivery systems. EVs are detected in a wide range of biological fluids, including blood, urine, saliva, cerebrospinal fluid (CSF), milk, and tumor exudates, enabling minimally invasive sampling for both research and clinical applications (Raposo and Stoorvogel, 2013; Yáñez-Mó et al., 2015). This section provides an overview of EV history, biology, classification, cargo composition, and mechanisms of interaction, highlighting their relevance to health and disease.

### 2.1 History and biology

The study of EVs dates back to the 1940s, when Chargaff and West first identified blood-clotting particles that were later recognized as functional cellular components. In the 1960s, Peter Wolf described ‘platelet dust’ in biological fluids, initially thought to be cellular debris (Hargett and Bauer, 2013). It was not until the 1980s that EVs were recognized as active participants in cellular processes, capable of excreting unwanted proteins and influencing biological functions (Hargett and Bauer, 2013; Chatterjee et al., 2020). Research in the 1990s expanded their known roles in coagulation and enzymatic activity, and modern studies have firmly established EVs as mediators of immune regulation, tissue repair, and disease progression (Doyle and Wang, 2019; Hargett and Bauer, 2013; Couch et al., 2021). EVs are inherently heterogeneous in size, composition, and function, reflecting both cell type and physiological or pathological context. They facilitate intercellular communication *via* multiple mechanisms, including direct membrane fusion, receptor-ligand interactions, and endocytosis *via* macropinocytosis or phagocytosis. In addition to signaling, EV-mediated disposal of metabolic byproducts, damaged organelles, and misfolded proteins helps maintain cellular homeostasis (Kostallari et al., 2021; Ginini et al., 2022). These features underscore their dual roles in physiological maintenance and disease pathogenesis.

### 2.2 Classification and biogenesis

**FIGURE 1:**
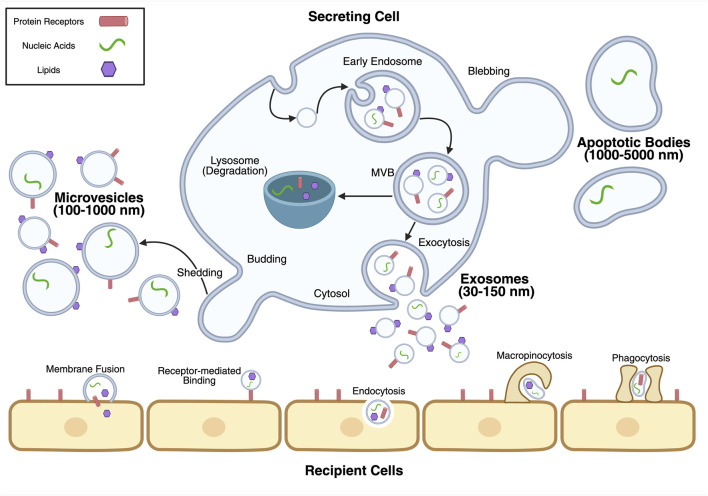
Biogenesis, release, and uptake pathways of EV. EV subtypes and their formation within secreting cells, along with uptake mechanisms by recipient cells. Abbreviation: MVB, multivesicular body.

The general classification of EVs is broadly based on their size, cellular biogenesis, and molecular composition, resulting in three historically described and operationally used EV categories: exosomes, microvesicles, and apoptotic bodies, while acknowledging that biogenesis-specific assignment is often not directly demonstrated in many studies (Figure 1) (Coughlan et al., 2020).

#### 2.2.1 Exosomes

The smallest EV subtype, typically ranging from 30 to 150 nm (nm) in diameter. They are formed within the endosomal compartment of the cell, which primarily comprises early endosomes, late endosomes (also known as multivesicular bodies, or MVBs), recycling endosomes, and lysosomes (Doyle and Wang, 2019). The biogenesis pathway of many EV types shares an initial dependence on Endosomal Sorting Complex Required for Transport (ESCRT)-regulated membrane remodeling. However, specific pathways diverge among exosomes, microvesicles, and apoptotic bodies, beginning with plasma membrane folding to form early endosomes, followed by inward budding of the late endosomal membrane to form MVBs (Doyle and Wang, 2019; Teng and Fussenegger, 2020). After this initial uniform phase, exosomes are synthesized *via* the endosomal pathway and released *via* exocytosis into the extracellular space, ultimately reaching the plasma membrane. Exosomes are rich in tetraspanin proteins, such as CD9, CD63, and CD81; ESCRT proteins, including Alix and TSG101; and lipids, including cholesterol and sphingomyelin (Doyle and Wang, 2019; Alptekin et al., 2022). Exosome cargo (consisting of proteins, lipids, mRNAs, and miRNAs) has been well studied and characterized for its ability to reflect the physiological or pathological state of the parent cell (Zhang Y. et al., 2019).

#### 2.2.2 Microvesicles (ectosomes)

These are large EVs, ranging from 100 to 1,000 nm in diameter. Microvesicles are released into the extracellular space *via* direct outward budding of the cell membrane, exposing cytoplasmic contents to the extracellular space (Doyle and Wang, 2019; Juan and Fürthauer, 2018). Alterations in cytoskeletal dynamics and phospholipid redistribution regulate the biogenesis process (Clancy et al., 2021). Microvesicles contain diverse molecular cargo that is often similar to exosomes but is more commonly associated with processes such as coagulation, inflammation, and cancer progression (Doyle and Wang, 2019).

#### 2.2.3 Apoptotic bodies

The largest EV subtype, which ranges in size from 1,000 to 5,000 nm (Clancy et al., 2021). They are released upon the fragmentation of the apoptotic cell, a process that often occurs during apoptosis, and are subsequently engulfed by neighboring cells or phagocytosed by macrophages (Juan and Fürthauer, 2018). Apoptotic bodies can encapsulate cellular material, including organelles, chromatin, and nuclear fragments, thereby enabling them to contribute to immune modulation and tissue remodeling (Doyle and Wang, 2019; Gustafson et al., 2017).

### 2.3 Composition and cargo

The functional roles of EVs stem from their capacity to transport a broad spectrum of bioactive molecules. EV cargo composition is highly diverse and reflects the physiological state of the parent cell, contributing to its functional impact on various biological processes (Colombo et al., 2014; Kalluri and LeBleu, 2020). The EV cargo includes:

#### 2.3.1 Proteins

EVs are highly enriched in membrane-associated proteins, including tetraspanins, cytoskeletal components, ESCRT proteins such as TSG101, heat shock proteins such as HSP70 and HSP90, signaling molecules, and proteins involved in vesicle biogenesis (Kostallari et al., 2021). Many of these proteins serve as identifying markers for EVs, distinguishing different subtypes (Théry et al., 2018).

#### 2.3.2 Lipids

EV cargo also contains lipids, which play functional roles in the EV membranes’ structure, stability, curvature, and bioactivity. Specifically, EV membranes are rich in sphingolipids, ceramides, cholesterol, and phospholipids, which contribute to their biophysical properties and interactions with recipient cells (Skotland et al., 2017). The lipid composition of EVs is independent and unique from that of their cell of origin and, therefore, can regulate cellular signaling pathways upon uptake by the recipient cell. In neurological and lysosomal storage disorders, such lipid remodeling may encode disease-specific signatures relevant for both diagnosis and mechanistic insights (Skotland et al., 2017; Record et al., 2014).

#### 2.3.3 Nucleic acids

EVs carry a variety of nucleic acids, including mRNA, microRNA (miRNA), long non-coding RNA (lncRNA), and even DNA fragments. EV-transferred nucleic acids (mitochondrial and genomic DNA) can regulate gene expression in recipient cells (Kalluri and LeBleu, 2020; Batagov et al., 2011), suggesting a potential role in the transfer of genetic material and disease pathogenesis (Kahlert et al., 2014).

Cargo loading is a highly selective process influenced by cellular state and environmental factors such as oxidative stress or hypoxia (Zhang L. et al., 2020). The cargo sorting mechanism involves both ESCRT-dependent and ESCRT-independent pathways. In the ESCRT-dependent pathway, the ESCRT machinery initiates ubiquitination and recruits ESCRT proteins to endosomal membranes (Hessvik and Llorente, 2018). In ESCRT-independent pathways, lipid raft-mediated sorting, ceramide-dependent cargo selection, and tetraspanin-enriched microdomains assist in the targeted loading of specific biomolecules (Hessvik and Llorente, 2018; Trajkovic et al., 2008). RNA-binding proteins, such as hnRNPA2B1 and YBX1, also influence the selective packaging of miRNAs, which affects their regulatory potential in the target recipient cells (Villarroya-Beltri et al., 2013; Liu X. M. et al., 2021).

### 2.4 Mechanisms of EV uptake and interaction

The biological effects of EVs depend not only on their cargo but also on their mode of interaction and uptake by recipient cells, which determines specificity and downstream signaling outcomes (Mulcahy et al., 2014). Major uptake mechanisms include:

#### 2.4.1 Endocytosis

The most common processes for internalizing EVs are clathrin-dependent and/or independent endocytosis, macropinocytosis, and phagocytosis (Mulcahy et al., 2014; Mathieu et al., 2019). Specifically, clathrin-mediated endocytosis has been linked with the uptake of smaller EVs (exosomes). Larger EVs (microvesicles, apoptotic bodies) may be internalized *via* macropinocytosis or phagocytosis (Tkach and Théry, 2016).

#### 2.4.2 Direct membrane fusion

Involves the direct fusion of EVs with the plasma membrane of the recipient cell. This pH-dependent process is influenced by lipid composition, particularly the presence of phosphatidylserine, which facilitates membrane fusion (Parolini et al., 2009) and allows for the direct release of EV contents into the cytoplasm (Gonda et al., 2019).

#### 2.4.3 Receptor-mediated processes

EV surface molecules (integrins, tetraspanins, and phosphatidylserine-binding proteins) can bind to surface receptors on target cells without being internalized, activating downstream signaling pathways that mediate targeted uptake (Niel et al., 2018). The expression of these surface markers determines EV tropism and influences their functional specificity (Kumar M. A. et al., 2024).

EVs can also activate intracellular signaling cascades through ligand-receptor interactions (Raposo and Stoorvogel, 2013). When immune cell-derived EVs that carry immunomodulatory molecules such as cytokines, chemokines, and regulatory proteins bind to plasma membrane receptors, they can regulate immune responses (Robbins and Morelli, 2014) and promote cellular and tissue regeneration (Ginini et al., 2022). EVs can also exert biological effects without internalization through ligand-receptor interactions that activate signaling pathways, including focal adhesion kinase (FAK) and downstream Mitogen-Activated Protein Kinase/Extracellular Signal-Regulated Kinase (MAPK/ERK) signaling (Luga et al., 2012). EV-associated lipids, such as sphingolipids and ceramides, participate in signal transduction pathways that regulate cell apoptosis, proliferation, and survival (Trajkovic et al., 2008). In the context of RND, these signaling pathways are particularly relevant because they intersect with neuronal stress responses, lysosomal trafficking, autophagy regulation, and neuroinflammatory cascades that frequently drive disease progression and age-associated neurodegeneration. Defects in lysosomal function and the resulting lipid imbalances may further alter EV biogenesis, cargo composition, and receptor-mediated signaling capacity, thereby reshaping EV-driven intercellular communication. Collectively, these diverse mechanisms position EVs as both reflective biomarkers of cellular state and active mediators of intercellular communication, reinforcing their diagnostic and therapeutic potential.

## 3 EVs in rare neurological diseases

EVs are increasingly recognized as innovative tools for clinical translation, encompassing biomarker discovery, therapeutic delivery, and regenerative medicine applications (Quek and Hill, 2017). While EVs have been extensively studied in cancer, cardiovascular disease, and other common conditions, their application in RND remains comparatively underexplored, despite representing a critical unmet need. Much of the current clinical and translational understanding of EV is derived from studies in cancer and metabolic disorders such as diabetes (Ciferri et al., 2021), providing a valuable framework for extension into rare disease contexts.

RND comprises a heterogeneous group of disorders with primary involvement of the brain, spinal cord, peripheral nerves, or muscles, and collectively affects millions of individuals worldwide, despite an individual disease prevalence of approximately one in 2,000 (Reinhard et al., 2021; Castillon et al., 2022). These disorders are often difficult to diagnose and manage due to limited epidemiological data, low disease awareness, small patient cohorts, and restricted access to specialized care, resulting in substantial unmet clinical needs. EV-based approaches offer a promising translational strategy by enabling minimally invasive, longitudinal, and potentially disease-specific monitoring and intervention, features that are particularly relevant for RND in aging populations.

### 3.1 Standardized approaches for EV isolation and characterization

**FIGURE 2:**
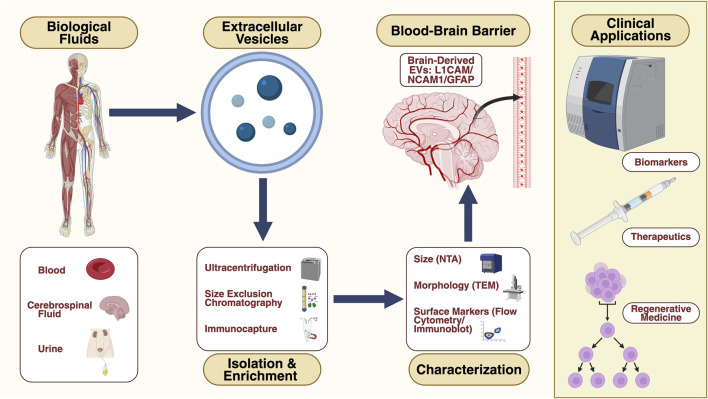
Overview of EV isolation, characterization, and clinical applications. EV can be isolated from blood, urine, or cerebrospinal fluid using ultracentrifugation, size-exclusion chromatography, or immunoaffinity-based methods. Characterization includes assessment of size, morphology, and surface marker expression. Brain-derived or brain-associated EV signals have been detected in peripheral biofluids in selected enrichment-based and preclinical studies, supporting potential applications in biomarker discovery, disease monitoring, therapeutic delivery, and regenerative medicine. Abbreviations: GFAP, glial fibrillary acidic protein; L1CAM, L1 cell adhesion molecule; NCAM1, neural cell adhesion molecule 1 (NCAM1); NTA, nanoparticle tracking analysis; TEM, transmission electron microscopy.

Rigorous standardization across all stages of EV research is essential, as outlined in the Minimal Information for Studies of Extracellular Vesicles (MISEV) 2023 guidelines (Welsh et al., 2024). Variables such as needle size, donor fasting, tube selection, and prompt sample processing must be carefully controlled to prevent platelet activation or hemolysis. Sample handling should minimize agitation and maintain appropriate temperature to preserve EV integrity. Potential contamination from platelets and lipoproteins must be assessed to ensure that EV profiles accurately reflect biological and disease-specific signals. EVs can be isolated using various validated methods, including ultracentrifugation, differential filtration, size-exclusion chromatography, polymer-based precipitation, immunoprecipitation, and microfluidic separation. Comprehensive characterization using nanoparticle tracking analysis (NTA), transmission electron microscopy (TEM), flow cytometry, and protein assays is crucial to ensure reproducibility and comparability across studies (Al Abdullah et al., 2024; Torrini et al., 2025). Adhering to these standardized protocols is critical for generating reliable and reproducible data, which forms the foundation for meaningful biomarker discovery and therapeutic development using EVs in RND. A schematic overview of EV isolation methods, characterization techniques, and their potential applications is presented in Figure 2.

### 3.2 EVs as biomarkers

EVs provide a noninvasive and dynamic window into disease biology, reflecting the molecular and cellular state of their cells of origin. Their biomarker utility spans three principal domains: diagnostic, prognostic (disease monitoring), and treatment response assessment (Figure 2) (Liu J. et al., 2021). By carrying disease-relevant cargo, EVs enable access to tissue-specific pathology that is otherwise difficult to obtain, a critical advantage for RND where invasive biopsies and repeated sampling are impractical. In aging-focused RND studies, key confounders should include age strata, concomitant medications, renal and hepatic function, inflammatory markers, and disease-modifying therapies that may alter EV secretion or cargo composition (Zhang Y.-T. et al., 2025).

#### 3.2.1 Diagnostic markers

EVs carry disease-specific cargo that mirrors the pathological processes of their parent cells (Kalluri and LeBleu, 2020). In cancer, tumor-derived EVs enable early detection of oncogenic mutations and tumor-specific surface proteins without invasive biopsies, a paradigm that is increasingly applicable to neurological diseases. CNS-derived EVs circulating in blood or CSF can serve as a minimally invasive source for detecting neurological pathology (Melo et al., 2015).

Timely diagnosis is especially crucial for RND, where rapid progression can result in severe morbidity or mortality. In selected preclinical and enrichment-based biomarker studies, EV-associated CNS signals have been detected in peripheral biofluids, supporting their potential utility for minimally invasive monitoring rather than establishing uniform blood-brain barrier (BBB) traversal across clinical settings (Sinha et al., 2018; Yan et al., 2024). However, interpretation depends strongly on methodology, including the EV population analyzed, the enrichment strategy used, contamination control, and normalization approach. For example, studies of putative neuronal EV in blood have commonly used immunocapture approaches based on neural-associated markers, but the specificity of these markers and the potential contribution of platelet, lipoprotein, or soluble protein contaminants remain important limitations. Across studies, normalization has varied by particle count, total protein, or input volume, and performance metrics have not been reported consistently, limiting direct comparison and clinical interpretability. Although direct biomarker evidence in RND remains comparatively limited, studies in common neurodegenerative disorders provide a translational context for EV-associated CNS pathology. In AD, EVs isolated from CSF or blood have been reported to contain amyloid-beta (Aβ) and tau species, while in PD, α-synuclein–associated EV populations have shown associations with disease state or severity in selected cohorts (Yan et al., 2024; Wang X. et al., 2024; Sinha et al., 2018). In GD, exosomes from iPSC-derived astrocytes reflect glucocerebrosidase dysfunction, and plasma EV from patients contain GCase- and lysosomal-associated cargo, linking rare disease biology to translational insights from synucleinopathies (Aflaki et al., 2020; Tatiana et al., 2020). Similarly, EV-associated TAR DNA-binding protein 43 (TDP-43) has been investigated as a potential biomarker in amyotrophic lateral sclerosis (Dellar et al., 2025). Collectively, these findings support the promise of EV-based liquid biopsies in RND and underscore the need for standardized capture, normalization, and validation frameworks.

#### 3.2.2 Prognostic markers (real-time monitoring)

EV profiles evolve in tandem with the disease course, making them useful as temporal indicators of neurodegeneration and treatment exposure. Beyond their diagnostic capabilities, EV can also provide real-time monitoring of disease progression and long-term prognosis, which is vital for an aging population with neurological diseases and comorbidities. As cells change during disease progression, the molecular composition of the EVs they release also changes. Observing the continuous changes in the dynamic profile of EVs provides a valuable tool for longitudinal tracking and risk stratification of the disease state, offering a unique potential prognostic metric for rare diseases with limited options for tracking disease progress (Doyle and Wang, 2019; Pegtel and Gould, 2019). In cancer, for example, EV-associated PD-L1 is correlated with immune evasion and poor prognosis in cancer patients (Chen et al., 2018). Consistent with the limited but growing RND literature, longitudinal studies in genetically defined neurodegenerative comparator disorders such as Huntington’s disease (HD) have identified changes in EV cargo, particularly mutant huntingtin (mHTT) protein levels, that are linked to disease state and symptom severity (Ananbeh et al., 2021). EVs are also implicated in the spreading of neurotoxic misfolded proteins, such as α-synuclein, tau, prion protein, and amyloid precursor protein throughout the CNS, potentially contributing to the progression of diseases like PD, AD, and Creutzfeldt-Jakob disease (CJD) (Properzi et al., 2015; Khadka et al., 2023). In multiple system atrophy (MSA), another synucleinopathy, elevated EV-associated oligomeric α-synuclein levels have been proposed as a marker of disease progression (Yu Z. et al., 2020). These observations highlight EVs as noninvasive, temporally sensitive indicators of disease evolution, well-suited to personalized monitoring in aging rare disease populations.

#### 3.2.3 Treatment response biomarkers

EVs also provide a window into therapeutic efficacy. Changes in EV cargo reflect cellular responses to treatment, allowing clinicians to assess therapeutic success or resistance (Liu J. et al., 2021). In cancer, a reduction in tumor-derived EV post-treatment indicates a positive response, whereas persistent oncogenic EV cargo suggests resistance (Del Re et al., 2019). In neurodegenerative diseases, EV monitoring tracks responses to gene therapies and disease-modifying interventions by measuring levels of pathological proteins (Raghav et al., 2022). Similarly, in lysosomal storage disorders, the effects of enzyme replacement therapy (ERT) are measurable by normalization of EV cargo in patient plasma, supporting clinical decision-making (Gleason et al., 2021). Thus, EV-based monitoring offers a robust, noninvasive approach for optimizing and personalizing therapy in RND.

Across diagnostic, prognostic, and treatment-response applications, the principal barriers to clinical translation are methodological rather than conceptual. Key sources of variability include differences in biofluid source, EV isolation method, enrichment strategy, contamination by platelets, lipoproteins, or soluble proteins, and inconsistent normalization by particle number, total protein, or input volume. In addition, many studies remain limited by small cohort size, cross-sectional design, and incomplete reporting of diagnostic performance metrics such as sensitivity, specificity, positive and negative predictive values (PPV and NPV), and area under the receiver operating characteristic curve (ROC-AUC). For RND, where cohorts are inherently small and biologic heterogeneity is high, these limitations make standardization and external validation especially important. Few examples on these biomarker applications of EVs for rare and common neurological conditions are summarized in Table 1.

**TABLE 1:** Representative EV biomarker applications in rare and comparator neurological disorders.

Disease	EV population/Fraction	Biofluid	Candidate cargo	Isolation/Enrichment approach	Intended context of use	Key limitation
Gaucher disease (GD)	Plasma EVs and exosomes from iPSC-derived astrocytes	Plasma and iPSC culture supernatant	GCase and lysosomal-associated proteins	Patient plasma EV isolation and iPSC-derived astrocyte exosome analysis	Diagnostic/Mechanistic biomarker	Limited cohort depth, workflow heterogeneity, and translational linkage to CNS pathology remain indirect
Niemann-Pick type C disease (NPC)	MSC-derived EVs in preclinical systems	Brain and peripheral tissues in model systems	Inflammation-related cargo and therapeutic response-associated signals	Preclinical EV treatment studies	Treatment response/Translational proof-of-concept	Primarily therapeutic rather than biomarker-focused evidence and limited human validation
Amyotrophic lateral sclerosis (ALS)	Disease-associated EV populations	Biofluids	TDP-43 aggregates	EV isolation from patient biofluids	Diagnostic biomarker	Need for standardized EV fraction definition and cohort validation
Huntington’s disease (HD)	EV cargo associated with disease state	Biofluids	Mutant huntingtin (mHTT)	Longitudinal EV cargo profiling	Prognostic/Disease monitoring	Longitudinal datasets remain limited, and thresholds for clinical use are undefined
Multiple system atrophy (MSA)	EV-associated α-synuclein populations	Biofluids	Oligomeric alpha-synuclein	EV biomarker analysis in selected cohorts	Disease monitoring/Progression marker	Cohort size and assay reproducibility remain limiting
Alzheimer’s disease (AD)	CSF- and blood-derived EVs	Blood and CSF	Amyloid-β; tau	EV isolation from CSF or blood	Diagnostic/Disease-state biomarker	Reported performance depends on assay design, and direct cross-study comparison is difficult
Parkinson’s disease (PD) (Observational)	Putative neuronal EVs	Blood and CSF	α-synuclein	Neural-marker-based immunocapture and EV analysis	Diagnostic/Severity association	Neuronal capture specificity, contamination control, and normalization vary across studies
PD - exenatide trial (Interventional)	Neuronal exosomes	Blood	Brain insulin signaling markers and Akt/mTOR-related signals	Blood-based neuronal exosome analysis	Treatment response/Pharmacodynamic readout	Clinical utility depends on robust enrichment specificity and independent validation

Abbreviations: CSF, cerebrospinal fluid; EVs, Extracellular vesicles; GCase, Glucocerebrosidase; MSC, mesenchymal stem cell.

### 3.3 Therapeutic applications

EVs hold therapeutic potential for aging individuals with RND by modulating neurodegenerative processes, delivering neuroprotective or regenerative cargo, and counteracting tissue decline (Putri et al., 2025; Ganesh et al., 2025). Notably, aging alters EV composition, secretion, and function, emphasizing the need for age-adapted therapeutic strategies (Eitan et al., 2017; Hooten et al., 2022).

#### 3.3.1 EVs as delivery vehicles for therapeutics

EVs possess several attractive features as delivery platforms, including biocompatibility, low immunogenicity, and reported capacity in selected preclinical systems to traverse biological barriers relevant to CNS delivery. In AD, MSC-derived EVs loaded with miR-29b reduce tau hyperphosphorylation and mitigate neuroinflammation (Zhang X. et al., 2021). In PD, astrocyte-derived EVs enriched with GCase restore lysosomal function and reduce α-synuclein accumulation, particularly in *GBA1*-associated PD (Haney et al., 2015). In Niemann-Pick type C (NPC), MSC-EV reduces inflammation in the brain and peripheral tissues in preclinical models (Geberhiwot et al., 2018; Hoecke et al., 2021). Together, these studies support the preclinical potential of EV-mediated CNS delivery, but direct evidence in RND-specific systems and clinical settings remains limited.

#### 3.3.2 Engineering EV for targeted treatment

EV can be engineered to enhance specificity and efficacy. In ALS, an engineered EVs carrying a small interfering RNA (siRNA) targeting mutations in the superoxide dismutase type 1 (SOD1) gene successfully reduced toxic protein aggregation in preclinical models (Guo et al., 2026). Additionally, targeting EVs modified with BBB-penetrating ligands has been shown to improve the delivery of therapeutic agents to the CNS. For example, EVs conjugated with rabies virus glycoprotein (RVG) peptides have been used to deliver siRNA targeting β-site amyloid precursor protein-cleaving enzyme 1 (BACE1), a key enzyme in amyloid-beta (Aβ) production, demonstrating efficacy in AD models (Yuyama et al., 2015). Such engineering approaches enable precision therapies for RND, for which treatment options are limited.

### 3.4 EVs in regenerative medicine

EVs play a multifaceted role in regenerative medicine by mediating intercellular communication, promoting tissue repair, and regulating stem cell behavior. Through their bioactive cargo, EVs support wound healing, modulate inflammatory and regenerative signaling pathways, and enhance both stem cell and iPSC-based therapeutic strategies. Collectively, these functions position EVs as versatile, cell-free tools with growing potential for personalized, age-adapted regenerative therapies. For RNDs that are characterized by irreversible tissue damage and limited regenerative capacity, EV-mediated regenerative approaches may complement enzyme replacement, gene therapy, or small-molecule treatments, particularly in aging patients.

#### 3.4.1 Tissue repair and regeneration

EVs play a central role in tissue repair and regeneration by orchestrating intercellular communication and key biological processes, including the resolution of inflammation, angiogenesis, and cellular differentiation. Stem cell-derived EVs, particularly MSC-EVs, transfer bioactive molecules to recipient cells, thereby enhancing wound healing and tissue remodeling. This process has been demonstrated in fibroblasts to promote migration and proliferation, thereby accelerating wound closure (Phinney and Pittenger, 2017; Zhang B. et al., 2020). These regenerative mechanisms are especially relevant in aging populations, who exhibit diminished healing capacity and are more vulnerable to complications from neurodegenerative diseases. By restoring tissue integrity and modulating chronic inflammation, EV-based therapies may counteract age-related declines in repair processes and help manage multi-system involvement seen in rare disorders (Ganesh et al., 2025).

#### 3.4.2 Stem cell biology and therapy

EVs contribute to stem cell regulation by modulating cell fate decisions, self-renewal, and differentiation. Stem cell-derived EVs can carry cargo that regulates key signaling pathways, such as Wnt/β-catenin, Notch, and PI3K/Akt, which are crucial for maintaining and differentiating stem cells (Nawaz et al., 2015). Importantly, EVs can replicate many of the therapeutic benefits of stem cells without the associated risks of direct transplantation, such as immune rejection or tumorigenicity (Bahmani and Ullah, 2022). In preclinical models, stem cell-derived EVs have shown promise in improving functional recovery in conditions such as myocardial infarction, stroke, and osteoarthritis (Karnas et al., 2023). These cell-free approaches hold significant therapeutic potential for elderly patients, who often cannot undergo invasive procedures or tolerate immunosuppressive therapies. By improving regenerative signaling and reducing systemic inflammation, stem cell-derived EVs may help mitigate progressive degeneration and functional decline in aging individuals with rare pathologies (Ganesh et al., 2025).

#### 3.4.3 Induced pluripotent stem cells (iPSCs)-derived EVs

iPSC-derived EVs are increasingly recognized for their role in maintaining pluripotency, guiding differentiation, and regulating immune responses. These EVs contain key pluripotency-related factors, including Oct4, Sox2, and Nanog, that influence somatic cell reprogramming and sustain the self-renewal properties of iPSCs (Wang A. et al., 2025). iPSC-derived EVs can modulate epigenetic modifications in target cells by transferring non-coding RNAs and chromatin-remodeling proteins, thereby influencing lineage specification (Wang A. et al., 2025; Mannavola et al., 2019). iPSC-derived EVs also deliver neurotrophic factors and anti-inflammatory miRNAs, such as brain-derived neurotrophic factor (BDNF), glial cell line-derived neurotrophic factor (GDNF), miR-21, and miR-146a, that support neuronal survival and immune regulation (Moretti et al., 2025; Yu M. et al., 2025). These properties make them attractive candidates for regenerative therapies in RND, where both neurodegeneration and chronic inflammation are central.

In addition to therapeutic applications, EVs from patient-specific iPSCs offer a personalized, non-immunogenic platform for drug delivery and gene editing. Engineered iPSC-EVs can transport therapeutic molecules, including CRISPR/Cas9 and siRNAs, enabling targeted correction of disease-causing mutations (Wang A. et al., 2025). This precision is particularly beneficial for older adults with rare genetic disorders, where systemic treatments may lack specificity and cause adverse effects. Moreover, iPSC-EVs have demonstrated utility in disease modeling and biomarker discovery. In PD and AD, iPSC-derived neurons release EVs containing disease-relevant miRNAs and misfolded proteins, offering insights into pathogenesis and the potential for early diagnosis (Vandendriessche et al., 2020; Jacquet et al., 2021; Aulston et al., 2019). As the global population ages, the diagnostic, regenerative, and precision-medicine applications of iPSC-derived EVs are poised to play an increasingly important role in addressing the complexity of RND.

## 4 Brain-derived extracellular vesicles as biomarkers and therapeutic tools

Brain-derived extracellular vesicles (BDEV) are increasingly recognized as powerful tools for understanding disease mechanisms, identifying biomarkers, and developing therapeutic strategies in RND (Xu et al., 2024). These EVs provide a minimally invasive source for longitudinal monitoring, facilitating broader patient inclusion, a crucial advantage for rare disease studies with limited sample sizes (He et al., 2024). Their accessibility from peripheral blood and ability to carry CNS-specific molecular cargo make BDEV particularly promising for both biomarker discovery and therapeutic applications in RND.

### 4.1 BDEV in neurodegeneration

Emerging evidence indicates that BDEV plays a central role in the pathogenesis of neurodegenerative diseases (You and Ikezu, 2019). In AD, neuron- and astrocyte-derived EV subpopulations have been enriched from plasma using antibodies targeting putative neural markers such as L1 cell adhesion molecule (L1CAM), neural cell adhesion molecule 1 (NCAM1), and glial fibrillary acidic protein (GFAP) (Al Abdullah et al., 2024). However, the specificity of these targets requires careful interpretation. L1CAM, widely used for neuronal EV enrichment, predominantly exists as a soluble cleaved protein in plasma and CSF rather than a membrane-bound EV antigen, and is also expressed in non-CNS tissues (Norman et al., 2021; Kadam et al., 2025). NCAM1 is expressed in multiple cell types, including natural killer cells and myocytes, whereas GFAP, an intracellular astrocytic protein, is less suitable for surface-based capture (Torrini et al., 2025). Despite these constraints, such approaches have yielded biologically relevant cargo profiles, highlighting the importance of complementary validation strategies. Evidence supporting CNS origin is strengthened by co-enrichment of neuronal cargo markers (e.g., neurofilament light chain, SNAP25, βIII-tubulin), use of isotype and depletion controls, and demonstration that captured material co-fractionates with canonical EV tetraspanins rather than soluble protein fractions (Norman et al., 2021; Kadam et al., 2025). Inclusion of negative controls, such as platelet (CD41) and erythrocyte (CD235a) markers, further reduces the likelihood of contamination.

In RND studies specifically, where systemic disease involvement, such as hepatosplenomegaly in GD or peripheral neuropathy in leukodystrophies, may influence peripheral expression of enrichment targets, terms such as putative BDEV or neural markers-associated EVs are preferable unless CNS origin is confirmed through multiple orthogonal approaches. With these methodological considerations in mind, and when validated using the approaches described above, putative BDEV fractions can contain pathogenic proteins, regulatory RNAs, and other vesicular cargo relevant to disease mechanisms, although the specificity of peripheral enrichment remains dependent on marker selection and orthogonal validation. Multi-omics analyses of BDEV reveal molecular signatures that correlate with disease progression and may serve as critical biomarkers for monitoring and diagnosis (Huang et al., 2022). Clinical studies also show that neuronal exosomes in the blood can serve as valuable biomarkers of the effects of drugs targeting the brain. In the Exenatide PD trial, these exosomes detected changes in brain insulin and Akt/mTOR signaling, demonstrating their value for measuring CNS responses from a blood sample (Athauda et al., 2019). Collectively, these findings highlight the potential of BDEV not only as mechanistic indicators of neurodegeneration but also as accessible biomarkers for early detection, disease monitoring, and the development of targeted therapeutic strategies in RND.

### 4.2 Clinical applications and study design considerations

Integrating BDEV into clinical studies requires careful consideration of study design, patient selection, and analytical approaches. Comprehensive patient metadata, including disease phenotype, stage, medications, environmental exposures, lifestyle factors, and family history, is essential to disentangle the genetic and environmental contributions to EV signatures. This is particularly important in RND, where patient cohorts are small and subtle molecular differences can be critical for biomarker discovery.

Longitudinal study designs allow tracking of temporal changes in BDEV cargo, providing insights into disease progression, early detection, and therapeutic response. Family-based or case-control designs further improve the specificity and sensitivity of biomarker identification by controlling for shared genetic and environmental factors. Standardization of sample collection, EV isolation, and molecular characterization is vital for reproducibility across studies.

For rare disease cohorts, a feasible longitudinal design may include a baseline followed by sampling at 6–12-month intervals, for at least 24 months. Mixed-effects longitudinal modeling, a statistical approach that analyzes both fixed effects (factors that are constant across individuals) and random effects (individual-specific variations), can be used to account for repeated measures, inter-individual heterogeneity, and missingness that are common in ultra-rare populations (Bilgel et al., 2016).

Integrating multi-omics approaches, including proteomics, transcriptomics, and lipidomics, enables comprehensive profiling of BDEV and reveals novel disease markers and potential therapeutic targets. Even before focusing exclusively on BDEV-specific multi-omics, integrating whole-sample omics data, such as blood or plasma proteomics or transcriptomics, provides essential context for identifying brain-derived signals within the broader systemic landscape. Comparing molecular profiles across total biofluid omics, BDEV-enriched fractions, and EVs from other cellular sources allows correlation analyses that help distinguish brain-specific alterations from peripheral or whole-body changes. This approach strengthens both the biological interpretation and the specificity of BDEV-associated biomarkers (Cohn et al., 2021).

Collaborations with rare disease registries and multicenter networks increase sample sizes, enhance statistical power, and improve external validity. These collaborative frameworks also facilitate establishing reference ranges for BDEV biomarkers, which are essential for distinguishing disease-specific alterations from normal variability. By integrating careful study design, rigorous standardization, multi-omics profiling, and collaborative strategies, BDEV offers significant potential as a minimally invasive tool for diagnosis, monitoring, and guiding therapy in RND. This approach bridges the gap between basic research and clinical application, enabling the translation of BDEV findings into meaningful patient care.

### 4.3 Regulatory considerations

Regulatory requirements for the clinical translation of EV-based biomarkers and therapeutics depend on their intended application, such as use as a biological product, a drug delivery system, a cell-free therapy, or a biomarker assay.

In the United States, the Food and Drug Administration (FDA) oversees EV-based products through existing regulatory frameworks for biologics, drugs, and *in vitro* diagnostics, as no EV-specific guidance has been established to date. Researchers must align with these established pathways while addressing challenges related to analytical validation, EV heterogeneity, and manufacturing controls. Therapeutic EVs derived from human cells are regulated by the FDA as cell-free biologics, primarily under the oversight of the Center for Biologics Evaluation and Research (CBER) and the Office of Tissues and Advanced Therapies (OTAT). Unlike cell-based therapies, EVs do not carry risks associated with live cells. When engineered to deliver therapeutic cargo, EVs may be classified as combination products and are subject to oversight by both CBER and the Center for Drug Evaluation and Research (CDER). For EV-based therapies, the FDA evaluates regulatory oversight based on the primary mode of action, i.e., the biological activity of the vesicle and its cargo, as outlined in the *Classification of Products as Drugs and Devices and Additional Product Classification Issues* (FDA, 2017).

EV-based biomarkers can be regulated as *in vitro* diagnostic devices (IVDs) under the Center for Devices and Radiological Health (CDRH). Depending on their risk and intended use, these assays may follow the 510(k) pathway, *De Novo* classification, or Premarket Approval (PMA), particularly if they guide treatment decisions or monitor therapeutic responses in clinical trials. The FDA’s Biomarker Qualification Program (BQP) oversees the qualification of EV-based biomarkers for RND and other indications. Under the Biomarker Qualification: Evidentiary Framework (FDA, 2018b), each biomarker must have a clearly defined Context of Use (COU), for example, diagnostic, prognostic, predictive, pharmacodynamic, or monitoring, since qualification is specific to its intended application. To qualify EV-based biomarkers, the FDA requires strong evidence in three key areas:

#### 4.3.1 Analytical validation

Demonstrates that the EV assay reliably and reproducibly measures the intended analyte. Key performance characteristics include accuracy, precision, sensitivity, specificity, reproducibility, and defined limits of detection and quantification. Controlling pre-analytical variables, such as sample collection, handling, and storage, is critical to ensure consistency across operators, instruments, and study sites, in accordance with FDA guidance on Bioanalytical Method Validation (FDA, 2018a).

#### 4.3.2 Clinical validation

This establishes a meaningful statistical and biological relationship between the EV biomarker and relevant clinical endpoints. This includes associations with disease presence, severity, progression, or response to treatment. Performance metrics such as sensitivity, specificity, PPV, NPV, and ROC-AUC should be evaluated. When feasible, validation should be confirmed across independent patient cohorts, following recommendations in Rare Diseases: Common Issues in Drug Development (FDA, 2019).

#### 4.3.3 Clinical utility and qualification

This demonstrates that the use of the EV biomarker meaningfully improves decision-making in drug development or clinical care. FDA qualification is context-specific; approval for one defined use does not imply endorsement for other applications.

EV-based therapeutics, even as cell-free products, must meet robust safety and manufacturing standards similar to those applied to biologics, including guidance from gene and cell therapy regulations. Developers must thoroughly characterize the source material, establish consistent and scalable manufacturing processes, validate identity and potency assays, and perform rigorous purity and safety testing. Safety evaluation requires assessment of immunogenicity, off-target effects, biodistribution, potential horizontal gene transfer, and toxicology, and may include long-term monitoring for CNS-targeted therapies. Since no EV-specific regulatory guidance currently exists, early engagement with the FDA is recommended to align EV-based products with established regulatory frameworks, clarify analytical and clinical validation requirements, and ensure compliance with applicable safety, manufacturing, and quality standards.

## 5 Advantages and limitations of EV

EVs represent a versatile platform for advancing diagnostics and therapeutics in RND, particularly in aging populations where conventional sampling is often limited. The extensive utility of EVs across both rare and common neurological pathologies is further illustrated in Table 2. Their endogenous cargo and intercellular communication capabilities offer significant clinical potential, but fully realizing it requires careful consideration of both their advantages and the technical challenges.

**TABLE 2:** Current applications of EVs in rare and common neurological pathologies.

Neurological pathologies	Biological samples	Experimental model	Translational applications	Key findings	References
Rare conditions					
Amyotrophic lateral sclerosis (ALS)	Astrocyte-derived EVs	Astrocytes	Astrocyte EV-driven motor neuron toxicity	EVs from ALS astrocytes selectively induce motor neuron death, supporting EV-mediated propagation of neurotoxic signals that cause neurotoxicity and morphological changes.	Marton et al. (2023)
Batten disease (neuronal ceroid lipofuscinoses type 2, CLN2)	Genetically modified macrophage-derived EVs	Macrophages and CLN2 mouse	EV-based lysosomal enzyme therapeutic delivery	Tripeptidyl peptidase-1 (TPP1)-loaded EVs successfully reached the CNS, activated autophagy, improved neuronal survival, and reduced neuropathology *in vivo*.	El-Hage et al. (2023)
	Enzyme-loaded macrophage-derived EVs	Macrophages and CLN2 mouse	EV-mediated tripeptidyl peptidase-1 (TPP1) replacement therapy	EVs efficiently delivered functional TPP1 to lysosomes and the brain, reducing neuroinflammation and restoring lysosomal function compared with free enzyme administration in CLN2 mice.	Haney et al. (2019)
Huntington’s disease (HD)	Human fibroblast-derived EVs	Human iPSC-derived GABAergic neurons	Functional rescue of neurons *via* healthy donor EVs	Fibroblast-derived EVs restored GABAergic transmission and mitochondrial function, and corrected synaptic deficits in HD neurons.	Beatriz et al. (2023)
	Hydrophobically modified siRNA (hsiRNA)-loaded exosomes	Mouse-derived neurons	Exosomes deliver hsiRNA targeting Huntingtin (HTT) mRNA for gene silencing	hsiRNA-loaded exosomes achieved robust HTT mRNA/protein silencing in neuronal cultures and demonstrated therapeutic gene knockdown in mice.	Didiot et al. (2016)
Machado-Joseph disease (MJD)/spinocerebellar ataxia type 3 (SCA3)	iPSC-derived neuroepithelial stem cell (NESC)- and neural culture-derived EVs	Human iPSC-derived neural cells	Autophagy and oxidative stress pathways assessment	MJD EV cargo contained distinct autophagy and oxidative stress components that downregulated autophagy and antioxidant proteins in recipient neurons, implicating EVs’ role in spreading pathological proteins.	Mendonça et al. (2025)
​	Neuronal targeting bioengineered EVs	Mouse-derived cerebellar cell lines	Gene therapy carriers delivering targeted silencing RNA sequences	Engineered EVs carrying miRNA-based silencing sequences significantly reduced RNA expression *in vitro*/*in vivo*. Neuronal targeting peptide enhancements improved packaging and brain targeting.	Rufino-Ramos et al. (2023)
Multiple system atrophy (MSA)	Neuron-derived EVs	Human serum	EV biomarker for mTOR pathway target engagement of a CNS therapeutic	Neuronal EV markers detected no difference from sirolimus-mediated modulation of mTOR signaling in the brain compared to placebo in the clinical trial.	Pucha et al. (2023)
	Oligodendrocyte-derived enriched microvesicles (OEMVs)	Human plasma and Oligodendrocyte isolated from a transgenic mouse	Peripheral biomarker of impaired SNARE protein-mediated microvesicles secretion	Dysfunction of the SNARE protein complex reduced oligodendrocyte microvesicles release, potentially contributing to impaired glial-neuronal communication in the disease.	Yu et al. (2020)
Gaucher disease (GD)	Cultured HEK293T cell-derived EVs	Macrophages, neuroblastoma cells, and neurons	EV-mediated therapeutic enzyme replacement	HEK293T-derived EVs delivered glucocerebrosidase (GCase) restored enzymatic activity and reduced lipid accumulation in macrophages and neurons derived from induced pluripotent stem cells (iPSCs) of patients with neuropathic GD.	Janpipatkul et al. (2024)
	Engineered EVs	HEK293 cells	Targeted lysosomal enzyme delivery	Engineered EVs enabled selective endocytic delivery and restored lysosomal enzyme activity in recipient cells, demonstrating a potential novel approach for GD with neurological complications.	Do et al. (2019)
Niemann-Pick type C disease (NPC)	Mesenchymal stromal cell (MSC)-derived EVs	NPC1-deficient mouse	Systemic anti-inflammatory EV therapy administration	MSC-derived EVs significantly reduced neuroinflammation, foamy cell accumulation, and gliosis in NPC1-deficient mice.	Hoecke L. et al. (2021)
	Oligodendrocyte- and fibroblast-derived exosomes	Oligodendrocytes and human fibroblasts	Endogenous EV-mediated cholesterol efflux	Enhanced exosomes secretion due to NPC1 deficiency promoted clearance of stored cholesterol, identifying a compensatory lipid export pathway.	Strauss et al. (2010)
Prion disease/transmissible spongiform sncephalopathy (TSE)	Prion-infected cell conditioned media EVs	Mouse neuroblastoma cells	EV-mediated prion transmission, pathogenic signaling, and disease propagation	Pathogenic prion proteins (PrPSc) packaged into EVs facilitated the intercellular spread of infectious prions to new cells.	Vella et al. (2007)
Rett syndrome (RTT)	iPSC-derived brain organoid conditioned media EVs	Human brain organoids	Biomarker discovery, developmental signaling, and disease modeling	RTT organoid-derived EVs demonstrated early, disease-specific dysregulation of a miRNA cluster, altering neurodevelopmental pathways.	Bahram Sangani et al. (2024)
Spinal muscular atrophy (SMA)	Engineered HepG2-derived EVs	Human HepG2 hepatocellular, A549 carcinoma, and fibroblast cells	Therapeutic delivery vehicles for protein uptake	EVs efficiently delivered functional survival motor neuron (SMN) proteins to recipient cells, with intracellular localization to the nucleus, and formed gem-like structures, including in SMN-deficient human cells.	René and Parks, (2025)
​	Human and mouse serum-derived serum EVs	Human and mouse fibroblasts and serum	Biomarker reservoir for monitoring protein levels	SMN proteins are released in EVs, with EV-associated SMN levels reflecting intracellular expression and disease state, potentially linking EVs as a non-invasive SMA biomarker.	Nash et al. (2017)
Common conditions					
Alzheimer’s disease (AD)	Human neural stem cell (hNSC)-derived EVs	Transgenic AD mouse	Neuroprotective EV therapy	Systematically administered hNSC-EVs reduced amyloid pathology, neuroinflammation, and synaptic protein loss, as well as improved cognition.	Apodaca et al. (2021)
	Neuronal-derived blood EVs	Human plasma and serum samples	Predictive EV biomarker discovery platform	Elevated EV tau and Aβ species are found in AD patients, with levels that predict AD development up to approximately 10 years before clinical onset and diagnosis.	Fiandaca et al. (2015)
	Microglia-derived EVs	Transgenic mouse	EVs as vehicles for tau propagation	Microglial EVs facilitated tau spread, and inhibiting EV biogenesis significantly reduced pathological tau propagation, suggesting a potential therapeutic target.	Asai et al. (2015)
​	Engineered dendritic cell-derived EVs	Wild-type mouse	Targeted delivery vehicles for BBB-crossing siRNA delivery	Systemically injected EVs delivered functional siRNA to the brain and achieved robust neuron-specific gene knockdown without immune activation.	Alvarez-Erviti et al. (2011)
Epilepsy	Neuronal-derived EVs	Astrocytes and neurons; rodent epilepsy models	Pathogenic intercellular signaling	Neuronal EVs containing miRNA (miR-181c-5p) impaired astrocyte protein expression, reduced glutamate uptake, and increased epilepsy susceptibility in animal models.	Ma et al. (2024)
	Forebrain-derived EVs	Fischer 344 rats	Molecular readout profiling of altered miRNA composition	Significant downregulation of miRNAs in forebrain-derived EVs from epileptic rats linked miR-346 and miR-331-3p to GABAergic signaling and mTOR pathways involved in epileptogenesis.	Gitaí et al. (2020)
Multiple sclerosis (MS)	Plasma-derived EVs	Human plasma	Biomarker and immune function regulation discovery	Drug dosing of fingolimod increased Plasma-derived EVs concentration, altered EV sncRNA cargo, and inhibited lymphocyte activation, suggesting EVs might reflect treatment prognosis.	Sáenz-Cuesta et al. (2018)
	Engineered microglial cell-derived EVs	Myeloid cell cultures; experimental autoimmune encephalomyelitis (EAE) induced mice	Immune modulation and therapeutic delivery by EVs	EVs delivered therapeutic cargo, reflected immune activation, and induced an anti-neuroinflammatory phenotype *in vitro* that reduced clinical severity and contributed to leukocyte recruitment.	Casella et al. (2018)
	Pregnant and virgin mice-derived serum exosomes	Oligodendrocyte precursor cells; pregnant (late gestation) mice	Functional use of exosomes-mediated immunosuppression and neuroprotection	Pregnancy-associated exosomes suppressed autoreactive T-cell activation, while functional serum-derived EVs contributed to immune modulation and neuroprotection during late gestation in mice.	Williams et al. (2013)
Parkinson’s disease (PD)	Adipose-derived stem cell (ADSC) dopamine-conjugated EVs	Neurons and neuroblastoma cells; mouse model of PD	Autophagy-inducing EV therapeutic delivery and targeting strategy	Dopamine-functionalized EVs were able to cross the BBB and enhance brain targeting, while also inducing autophagy that reduced α-synuclein accumulation and dopaminergic degeneration.	Sul et al. (2024)
	Macrophage-derived EVs	Neuronal cells and activated macrophages; mouse PD model	Therapeutic drug delivery carriers	Macrophage-derived EV-mediated catalase brain delivery, reduced oxidative stress and neuroinflammation, and protected dopaminergic neurons better than free catalase and improved motor function.	Haney et al. (2015)
	Neural-derived EVs	Human plasma	Blood EV-based biomarker platform	Altered plasma levels of α-synuclein and DJ-1 proteins in neural-derived EVs distinguished PD patients from controls with moderate discrimination.	Zhao et al. (2019)
​	Neuron- and neuroglioma cell-derived EVs	Neuron- and neuroglioma cells	Pathologic protein propagation evaluation	Autophagy modulated EV-associated α-Synuclein oligomers release that were more preferentially internalized than free toxic oligomers, which induced aggregation in recipient neurons.	Danzer et al. (2012)
Other conditions					
Glioblastoma multiforme (GBM)	Glioma cell-derived EVs	Human glioma cells	Proteomic characterization of EV cargo	Epidermal growth factor receptor variant III (EGFRvIII) altered the proteome of EVs, revealing pro-invasive signatures and enriching tumor-promoting adhesion proteins.	Choi et al. (2018)
	Glioblastoma cell-derived EVs	Human cell cultures and serum samples	EV-based oncogenic signaling and biomarker detection	GBM EVs carried functional tumor RNAs and proteins that enabled angiogenesis and proliferation, which can be utilized for horizontal oncogenic transfer and noninvasive tumor detection.	Skog et al. (2008)
​	Glioma cell-derived membrane EVs	Human glioma cells; tumor-bearing mice	Functional intercellular transfer of oncogenic protein	GBM EVs transferred receptor proteins (EGFRvIII) to recipient cells, activating oncogenic pathways (MAPK/Akt), altering gene expression, and enhancing anchorage-independent growth.	Al-Nedawi et al. (2008)
Ischemic stroke	MSC-derived EVs	Astrocytes and neurons; rats with MCAo induced ischemic injury	Therapeutic carriers for miRNA transfer	MSC-derived EVs successfully delivered miRNA (miR-133b) to neurons and astrocytes in rats with middle cerebral artery occlusion (MCAo)- induced ischemic stroke, thereby enhancing angiogenesis and neurogenesis.	Xin et al. (2012)
	MSC-derived EVs	Adult male Wistar rats	Post-stroke cell-free therapeutic delivery	MSC-derived EVs significantly improved functional recovery in rodent MCAo models and promoted neurovascular plasticity after ischemic stroke compared with controls.	Xin et al. (2013)
Neonatal hypoxic-ischemic (HI) brain injury	MSC-derived EVs	Neonatal HI mouse pups	Therapeutic strategies targeting injury response and tissue regeneration	Treatment with MSC-derived EVs after injury in neonatal mice resulted in increased neuroprotection and improved functional recovery mediated by reduced glial activation and decreased neuronal density loss.	Kaminski et al. (2020)
	MSC-derived EVs	Rice-Vannucci neonatal mouse model	Therapeutic cell-free brain injury intervention	Promising utilization of MSC-EVs as a potential cell-free therapeutic intervention for neonatal HI brain injury, given their neuroprotective capacity to reduce neuronal cell death.	Sisa et al. (2019)
Neuropathic pain	MSC-derived EVs	Microglial cells; chronic constriction injury rat model	Therapeutic anti-inflammatory nanocarriers	MSC-derived EVs delivered miR-99b-3p to microglial cells, promoting autophagy and suppressing microglial activation and neuroinflammatory cytokines, thereby alleviating neuropathic pain hypersensitivity in rodent models.	Gao et al. (2023)
Peripheral nerve injury (PNI)	Schwann cell-derived EVs	Cultured neurons; rats with sciatic nerve injury	EV-mediated intercellular communication to promote regeneration	Schwann cell EVs promoted neurite outgrowth and axonal regeneration by transferring ribosomes and growth-associated molecules, leading to increased axon extension in rats after sciatic nerve injuries.	Lopez-Verrilli et al. (2013)
Traumatic spinal cord injury (SCI)	Engineered induced neural stem cell (iNSC)-derived EVs	Astrocytes; traumatic SCI mouse model	Biomaterial-based targeted delivery of siRNA	iNSC-derived EVs specifically delivered siRNA to SCI lesions, which suppressed inflammation, reduced apoptosis, and enhanced neuronal repair more effectively than non-engineered EVs.	Rong et al. (2023)
	Bone mesenchymal stem cell (BMSC)-derived EVs	Adult male Sprague-Dawley rats with SCI	Cell-free injury recovery therapeutic administration	BMSC-EVs reduced neuronal cell death and improved tissue preservation after SCI by suppressing pericyte migration, which enhanced motor function recovery.	Lu et al. (2019)
	MSC-derived EVs	Adult male Sprague-Dawley rats with SCI	Neuroinflammation modulating therapy	Human MSC-derived EVs reduced neuroinflammatory microglial activation, enhanced systemic immune modulation, and improved locomotor recovery and mechanical sensitivity.	Ruppert et al. (2018)
Traumatic brain injury (TBI)	Astrocyte-derived EVs	Mouse microglia; controlled cortical impact mouse model	Neuroinflammation signaling and microglial activation	Astrocyte-derived EVs carried inflammatory mediators (miR-873a-5p) that modulated microglial activation and reduced neuroinflammation and neurological deficits in mice following a TBI.	Long et al. (2020)

### 5.1 Advantages

EVs offer several key advantages for clinical applications in RND, stemming from their physiological origin, biocompatibility, and multifunctional properties. As cell-derived nanoparticles carrying endogenous biomolecules, EVs present a safer alternative to synthetic delivery systems, with reduced risk of immune rejection, toxicity, and adverse effects. For instance, mesenchymal stem cell (MSC)-derived exosomes have demonstrated immunomodulatory properties, making them particularly suitable for therapeutic applications (Phinney and Pittenger, 2017). Their natural composition, free from exogenous chemical modifications, contributes to low immunogenicity and a favorable safety profile (Lener et al., 2015). A particularly compelling potential advantage of EVs is their reported ability, in selected experimental systems, to cross biological barriers, including the BBB, a significant obstacle to treating neurological disorders. Although much of this evidence derives from common neurodegenerative and experimental CNS models rather than RND-specific systems, studies have shown that EV can traverse the BBB through mechanisms such as transcytosis, receptor-mediated endocytosis, and direct membrane fusion (Ramos-Zaldívar et al., 2022; Matsumoto et al., 2017). Neural stem cell-derived EVs, for example, have successfully delivered therapeutic cargo to target brain cells (Yang et al., 2015). Further, surface modification or ligand engineering can enhance tissue-specific targeting and delivery precision. Compared with CSF-based biomarkers and some imaging-dependent approaches, EV-based assays may enable more frequent and less invasive sampling, which is especially attractive for geographically dispersed RND cohorts.

### 5.2 Limitations

Despite their advantages, EVs face several practical and technical challenges that limit their widespread clinical use. One major obstacle is the isolation and purification process. Standard methods, such as ultracentrifugation, size-exclusion chromatography, or precipitation, can yield variable purity and risk of contamination (Coughlan et al., 2020). Additionally, the small size and low abundance of EVs in biological samples complicate scalable collection and reproducible analysis, particularly in rare diseases.

The high heterogeneity of EVs, even from the same cell type or biological source, further complicates accurate profiling and makes cross-study comparisons challenging. In the context of RND, this challenge is amplified by EV heterogeneity at the individual patient level, where vesicles released from the same tissue can vary substantially in cargo composition depending on cellular state, disease stage, and microenvironment. Such intra-patient variability complicates the identification of stable, disease-specific EV biomarkers and limits the development of standardized diagnostic thresholds for rare and clinically heterogeneous conditions (Kowal et al., 2016). It also complicates the establishment of clinically meaningful cutoff values and the reproducibility of performance estimates across studies, particularly when different normalization strategies and enrichment workflows are used. As a result, the lack of standardized protocols for isolation and characterization hampers reproducibility and slows clinical translation (Théry et al., 2018; Kumar M. A. et al., 2024). To address these limitations, the development of universally accepted protocols and robust frameworks for isolation, characterization, scalability, and clinical translation of EVs is critical. Such standardization is essential not only for regulatory approval but also for ensuring reproducible implementation of EV-based diagnostics and therapeutics across multiple clinical centers.

Beyond technical and biological challenges, regulatory expectations themselves represent a significant barrier. The lack of EV-specific FDA guidance creates uncertainty about acceptable standards for identity, potency, purity, and comparability, particularly given EV heterogeneity. Regulatory requirements for batch-to-batch consistency, quantitative potency assays, and validated manufacturing controls are difficult to meet, especially for products derived from biological sources, where donor or cell-line variability can complicate consistency assessments.

Stringent evidentiary requirements apply to analytical and clinical validation, as well as to the clinical utility of biomarkers in general. While essential for patient safety, these requirements pose challenges for RND when patient populations are limited, and longitudinal samples are scarce. Regulatory hurdles can slow development timelines, increase costs, and deter commercial investment. Nevertheless, as regulatory science evolves and precedents emerge from early EV-based clinical programs, clearer expectations may accelerate translation (Verma and Arora, 2025). Continued dialogue between regulators, academic investigators, and industry stakeholders will be critical to balance standardization and feasibility, unlocking the future potential of EV-based drugs and biomarkers.

## 6 Prospects

EVs are increasingly recognized as precision tools for diagnosing and treating RND, offering both minimally invasive biomarkers and customizable therapeutic delivery platforms. Growing advancements in EV research and related technologies underscore their potential to enhance multiple aspects of neurological disease management (Quek and Hill, 2017). Looking ahead, the field must prioritize standardizing protocols for isolation, characterization, and molecular profiling of EVs to improve reproducibility and comparability across patient cohorts and disease types (Doyle and Wang, 2019). The development of robust, EV-specific regulatory guidance and harmonized methodological standards will be a critical future milestone, enabling consistent validation, cross-study comparability, and broader clinical adoption of EV-based technologies.

Future studies should also integrate EV research with emerging innovations, such as single-vesicle profiling and artificial intelligence–driven biomarker discovery, which hold promise for expanding the sensitivity and specificity of EV-based applications (Ma et al., 2023). Additionally, bioengineered or nanotechnology-enhanced EVs may further optimize targeting specificity, enhance vesicle stability, and improve therapeutic efficacy for CNS disorders (Wu et al., 2023). Ultimately, continued innovation, rigorous validation, and regulatory alignment will be essential for translating these versatile platforms into clinically meaningful diagnostics and therapies for patients with RND.

## 7 Conclusion

While direct clinical validation in RND remains early, convergent evidence from rare neurometabolic disorders and translational comparator studies in common neurodegenerative diseases supports the growing relevance of EV-based CNS biomarker and therapeutic strategies. In selected preclinical, enrichment-based, and peripheral biofluid studies, EV-associated molecular signals have provided access to disease-relevant biology that is otherwise difficult to sample longitudinally. Patient-derived EV and putative brain-associated EV fractions have shown promise for monitoring disease severity and therapeutic response. However, their interpretation remains constrained by source specificity, vesicle heterogeneity, and incomplete validation across independent cohorts. Beyond diagnostic applications, engineered EVs are emerging as candidate vehicles for targeted delivery of therapeutic molecules, including nucleic acids, enzymes, and small-molecule agents, but translational readiness in RND remains limited. Continued progress will depend on standardized isolation and characterization workflows, rigorous analytical and clinical validation, and careful alignment between mechanistic promise and clinically realistic contexts of use.
